# Beyond the Evidence of the New Hypertension Guidelines. Blood pressure measurement – is it good enough for accurate diagnosis of hypertension? Time might be in, for a paradigm shift (I)

**DOI:** 10.1186/1468-6708-6-6

**Published:** 2005-04-06

**Authors:** Cornel Pater

**Affiliations:** 1 Chippenham, UK

## Abstract

Despite widespread availability of a large body of evidence in the area of hypertension, the translation of that evidence into viable recommendations aimed at improving the quality of health care is very difficult, sometimes to the point of questionable acceptability and overall credibility of the guidelines advocating those recommendations.

The scientific community world-wide and especially professionals interested in the topic of hypertension are witnessing currently an unprecedented debate over the issue of appropriateness of using different drugs/drug classes for the treatment of hypertension. An endless supply of recent and less recent "drug-news", some in support of, others against the current guidelines, justifying the use of selected types of drug treatment or criticising other, are coming out in the scientific literature on an almost weekly basis. The latest of such debate (at the time of writing this paper) pertains the safety profile of ARBs vs ACE inhibitors.

To great extent, the factual situation has been fuelled by the new hypertension guidelines (different for USA, Europe, New Zeeland and UK) through, apparently small inconsistencies and conflicting messages, that might have generated substantial and perpetuating confusion among both prescribing physicians and their patients, regardless of their country of origin.

The overwhelming message conveyed by most guidelines and opinion leaders is the widespread use of diuretics as first-line agents in all patients with blood pressure above a certain cut-off level and the increasingly aggressive approach towards diagnosis and treatment of hypertension. This, apparently well-justified, logical and easily comprehensible message is unfortunately miss-obeyed by most physicians, on both parts of the Atlantic.

Amazingly, the message assumes a universal simplicity of both diagnosis and treatment of hypertension, while ignoring several hypertension-specific variables, commonly known to have high level of complexity, such as:

- accuracy of recorded blood pressure and the great inter-observer variability,

- diversity in the competency and training of diagnosing physician,

- individual patient/disease profile with highly subjective preferences,

- difficulty in reaching consensus among opinion leaders,

- pharmaceutical industry's influence, and, nonetheless,

- the large variability in the efficacy and safety of the antihypertensive drugs.

The present 2-series article attempts to identify and review possible causes that might have, at least in part, generated the current healthcare anachronism (I); to highlight the current trend to account for the uncertainties related to the fixed blood pressure cut-off point and the possible solutions to improve accuracy of diagnosis and treatment of hypertension (II).

## Introduction and magnitude of the background problem

Recent changes in definition and classification of blood pressure levels make hypertension, by far, the most commonly diagnosed condition in primary and secondary healthcare systems and projects the entity on the first place in terms of work load and prescribing cost.

"People with normal blood pressure by their 50 years of age are considered to run a 90% life-time risk for developing hypertension later during their lives"[1].

This statement puts in perspective the epidemic nature of hypertension and the growing concern of all societies in dealing with this outstanding public health problem, in developed as well as in developing countries.

There is little doubt that the American [1], the European [2], the British [3] and the WHO [4] guidelines on hypertension have the same, common goal of improving the quality of health care by changing the behaviour of providers and by improving the effectiveness of hypertension management in daily practice. Overall, current guidelines have become larger documents, apparently more comprehensive and increasingly evidence-based. Despite these obvious improvements, guidelines are hardly, if at all, implemented in clinical practice.

### The demographic and socio-economic profile of hypertension

The National Health and Nutritional Health Survey (NHANES) [5] data from 1999 to 2000 reported a 3.7-percentage point increase in the hypertension prevalence rate with more than 42% of hypertensives being not at all treated, almost 30% of them being unaware of their illness and 69% not being controlled. Reported control rates are even lower in European countries, only 8% on average [6]. Approximately 75 million adults (34% of the whole population) have blood pressure above 140/90 mmHg in five European countries (UK, Germany, France, Italy and Sweden) [7].

These disappointing figures are in sharp contradiction with the hypertension-related successes over several years before the survey.

Even worse, the recently published results of an epidemiologic inquiry to assess the hypertension burden and overall prevalence for the same period (1999 to 2000) in US [8] showed that, in fact, the total hypertension prevalence rate reached 31.3% amounting to at least 65 million adults in US having hypertension. This is an almost 30% increase of the frequently quoted figure for the magnitude of hypertension in terms of prevalence based on the NHANES III estimation of "50 million adults" with hypertension in the US [8].

Besides, the newly minted *prehypertension *category in the JNC VII-hypertension classification [1], introduced on fully justifiable reasons, ads further to the hypertension-related public health burden. In a sample of 3,488 persons of the same NHANES III survey, among people aged 20 years or older, 29% were hypertensive, 31% were prehypertensive and 39% were normotensive, with considerable greater percent of prehypertensives in men than in women (39.0% versus 23.1%) and in blacks as compared with whites (37.4% versus 32.2%) [9,10]. About 59 million American adults (29%) fall into the prehypertension category.

These data suggest that more than 60% of the American adults are either hypertensive or prehypertensive. Noticeably, the prehypertesives were 1.65 times more likely to have at least one other major risk factor (total cholesterol or overweight) than were those with normotension. Furthermore, in a simulation applied to a sample of 10,000 adults aged 25–74 years, the considerably high prevalence of prehypertensives in the higher age groups (two-thirds among people aged 45–65 years and 80% of those aged 65–74 years) indicated that prehypertension might account for 3.4% of hospitalisation, 6.5% of nursing home stays, and 9.1% of deaths [11].

These health-related alarming trends are ascribed to the increasing prevalence of obesity in the general population as well as to the aging, respectively, to the growing segment of elderly in the general population.

Interestingly, the two latter demographic aspects are results of increased life expectancy [12-14] in the context of "healthier lifestyles and/or better control rates associated with hypertension-related public health effectiveness and medical care quality improvement", a mix of factors termed: "higher control-survival-burden paradox" [5].

Paradoxically indeed, improved hypertension-focused medical care has simultaneously increased the burden on public health systems worldwide! Estimating further this burden in the American-context, the 23 million hypertensive adults estimated to take antihypertensive medication generate costs of about $15bn (€12bn), i.e., 10% of the country's total spending on drugs [15].

Estimated total direct and indirect cost of hypertension for 2005, as a result of its increased prevalence, is around $60 billion while, the total cost for cardiovascular disease and stroke for 2005 is estimated to be $393.5 billion [16].

According to the same source [16], hypertension was listed as a primary or contributing cause of death in about 261,000 of about 2.4 million US deaths in 2002, representing a 57% increase in death from hypertension over the past few decades.

The projected addition of some 30 million individuals aged 60 years or older in the next 20 years [17] as well as the widespread consensus and willingness to increase the number of those effectively treated for their hypertension, emphasizes the potential for escalating costs that may blow out of proportion and the seriousness of the hypertension-epidemic.

In this context, it is obvious that there is a tremendous need for wide-scope, systematically driven cost-effective prevention and treatment, and for more effective control strategies at all levels of healthcare systems.

However, a holistic, comprehensive strategic approach must not only target hypertension as pathological entity, but must take into account the wider environment in which hypertension is a major risk factor for cardiovascular disease and its interplay in the constellation of other, well-known modifiable risk factors such as: tobacco use, hypercholesterolemia, overweight/obesity, physical inactivity, diet and, to great extent and more often, associated diabetes mellitus [18-20].

### Diagnosing hypertension – sources of errors in blood pressure measurement

Blood pressure measurement is by far, the most commonly performed screening tests in medical practice and, because the act of measuring blood pressure is perceived as simple and straightforward it has also become the most commonly used "in house" self-employed test.

For only 20 years ago, the task of people willing to monitor their blood pressure was considerably easier, at least as far as the reference value for normal/abnormal blood pressure was concerned. It could simply be derived by everyone just by adding his/her age to 100 and consider the resultant value as cut-off point for threshold to abnormality. Advice from physician was eventually sought as to whether drug treatment was appropriate or not.

However, gradually, the age-standardized BP became obsolete and things started to be more complicated, not only for lay people interested in monitoring themselves but also for physicians and nurses. Accuracy of measurement itself has become an issue while adjustment to ever changing "target cut-off points" (reference ranges) is widely required.

So, the apparent user-friendliness of the blood pressure measurement techniques is made more questionable by the evolving changes in the definition, classification, diagnosis and management recommendations in the area of hypertension. As a consequence, both professionals as well a lay people wishing to continue to measure blood pressure are currently supposed to comply with guidelines for blood pressure measurement [21](see Additional file 1), if they are to believe what they are measuring.

### Observer bias

Despite the clear guidelines on blood pressure measurement technique, there seems to be large inter-observer variations, both among nursing staff and physicians as well as between the two groups. A questionnaire meant to focus this issue, encompassing 28 senior nurses and 55 health professionals from 28 different clinics in UK, highlighted considerable such variation leading consequently to inappropriate action [22-24]. A similar questionnaire carried out in Sao Paulo State on 105 professionals as to their compliance with the blood pressure measurement recommendations by Perloff et al. [21], showed that nursing staff abided by 40% of the recommended procedures while medicine teachers, physicians and residents abided by approximately 70% [25].

Conventionally, differences of 5 mmHg are considered clinically significant although, results derived from more recent trials such as VALUE (26) and ALLHAT [27] indicated that blood pressure differences of 2 to 4 mmHg might be clinically important. In this context, errors in blood pressure measurement in excess of 15 mmHg or more, as reported by Campbell et al. [28], are obviously leading to misclassification of hypertensive patients and inappropriate treatment.

What is more worrying, while in the same time at least partially explaining why such huge discrepancies may occur in practice, is the fact that gaps in the basic theoretic and practical knowledge seem to be common among interns and first-year family practice residents, supposed to have just acquired the skills for accurate blood pressure measurement [29]. An interesting observational study in this respect, carried out at the Westminster Medical School in London, showed that 33% out of 80 doctors in training grades/junior hospital doctors, acknowledged no formal education on how to measure blood pressure, a finding confirmed further by the poor accuracy in blood pressure measurement displayed by one third of the study group [30].

The human error impact is further compounded by variables such as: cuff selection and application, incorrect cuff positioning and rapid cuff deflation rate [20,31], inadequate rest period [31], digit bias and lack of repeated measurements (see Additional file 2).

### Faulty equipment

Adding considerably to the degree of "human error" in the area of blood pressure measurement is the universally poor state of the measurement devices available, their inaccuracy and the unreliability of the measurement results generated.

A study, aimed to assess the accuracy of calibration and evaluation of physical condition of 524 sphygmomanometers (mercury and aneroid) used in hospital settings or private medical offices, showed that 44% of the aneroid sphygmomanometers used in hospital settings and 61% of those used in private medical practices were found inaccurate. The magnitude of inaccuracy ranged from 4–6 mmHg in 32% to more than 13 mmHg in 7% of the tested devices. It was concluded that the mercury and aneroid sphygmomanometers showed inaccuracies of 21% versus 58% and unreliability of 64% versus 70%, respectively [32].

Another study, aimed at the assessment of maintenance and calibration of sphygmomanometers used in 231 English general practices, showed that 9.2% of the 1,462 sphygmomanometers tested gave readings that were more that 5 mmHg inaccurate and that one in 54 practices had an arrangement for maintenance and calibration of the measurement devices [33].

A British study carried out in 18 practices and 67 GP offices showed digit bias in systolic and diastolic readings to the nearest 10 mmHg [34].

### Lack of standardized blood pressure measurement devices

Because of its accuracy and reliability, the mercury sphygmomanometer is generally regarded as the gold standard against which all other devices for blood pressure measurement should be compared [35]. Unfortunately, due to the widespread concern that the mercury contaminates the environment, the mercury sphygmomanometers are about to be replaced largely with alternative equipment [36].

In contrast to the mercury sphygmomanometer that is least dependent on calibration and maintenance [37], the aneroid devices need calibration against a known standard (mercury manometer or non-mercury pressure meters) at six months interval. Failed calibration test implies the need to return the device to the manufacturer.

Lack of calibration and maintenance of the aneroid sphygmomanometers, as reported by numerous studies carried out in different parts of the world, makes them highly doubtful for routine use in medical practice, unless aggressive programs of maintenance and calibration will be implemented in timely manner in order to overcome the problems associated with inaccurate measurement of blood pressure [38].

A second type of alternative blood pressure measurement device is the electronic automated manometer. Such a device assesses the oscillations of pressure in the cuff during deflation. The point of maximal oscillation corresponds to the mean intra-arterial pressure. The systolic and diastolic pressures are computed on the basis of an algorithm, commonly known to be patent protected. This means, in turn, that algorithms used by different manufacturers vary from device to device [39]. Besides, these devices display a particular vulnerability in certain clinical circumstances, such as patients with arrhythmias and with stiff arteries. Likewise, they are prone to error because of the dependence of the cuff deflation rate to the heart rate. If the deflation is too rapid or the heart rate is too slow (e.g., in patients treated with beta-blockers), inaccurate blood pressure measurement is likely to occur [40].

Allegedly, most of these blood pressure measurement devices are subject to validation according to internationally accepted protocols of the Association for the Advancement of Medical Administration (AAMI) or the British Hypertension Society (BHS). The latest validation protocol has been issued by the European Society of Hypertension, slightly modified as compared to the two protocols mentioned above [41].

Disappointingly, however, commercially available automated devices that have passed validating study protocols still display significant inaccuracies in blood pressure measurement. Gerin et al. showed that blood pressure measurements with such devices were inaccurate by at least 5 mmHg in 20 to 38% of the individuals tested [42]. Schwartz et al. state that when using blood pressure monitors that meet the AAMI and BHS validation criteria, more than 50% of the persons tested may have average measurements that are in error by more than 5 mmHg [43].

Even worse is the fact that many of the most commonly used blood pressure monitors in clinical practice, in US [44], have never passed an AAIM certification and do not even need to do so as there are no regulatory requirements in this respect.

## Blood pressure variability and its assessment by ABPM

Assuming that successful precaution measures would be taken with regard to all the above mentioned sources of errors, the office blood pressure measurement would still not be an accurate reflection of an individual subject's true blood pressure and of its impact on the on that subject's long term outcome.

Apart from the imperfection of the Korotkoff technique as compared with the intra-arterial blood pressure measurements [45], just adding to the aforementioned potential sources of error, the great diurnal variability inherent in the blood pressure behaviour (see Additional file 3) is perhaps the major issue that makes office blood pressure measurement a screening test at best, and unqualifies it as a diagnostic test.

Ambulatory blood pressure measurement (ABPM), now in use since more than 25 years, has been gaining wide acceptance during the past few years. Current guidelines recommend use of the method in selected cases (see Additional file 4) [46-49], however, in the light of more recent data [50,51,63], ABPM is about to emerge as the method of choice for ensuring an accurate "first time" hypertension diagnosis, for refining cardiovascular risk stratification in most of the newly-diagnosed hypertensives and for the ability to confidently start or postpone pharmacologic treatment in a particular individual. Furthermore, ABPM allows for assessment of prognosis and therapeutic guidance.

**Figure 1:**
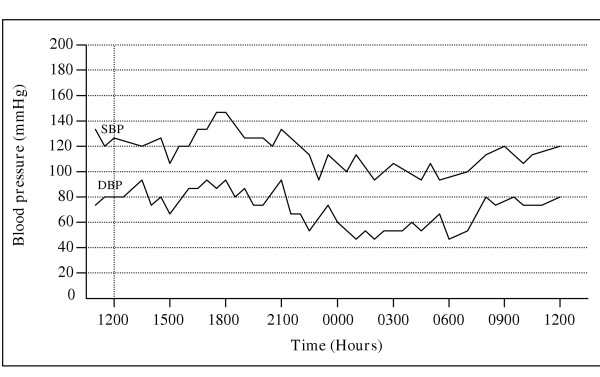
Blood pressure variability in healthy individual recorded by 24-hour ABPM.

A 24-hour ambulatory blood pressure curve (see Fig. 1) fully illustrates the large, diurnal intraindividual variability of the blood pressure and suggests that great differences may be encountered when comparing such data with the data collected from single or a few readings in the physician's office.

The great uncertainty that lies in casual blood pressure measurements, reflecting the biologic variations of this parameter, has been emphasized through Bayesian analysis [52] and a prospective trial of home blood pressure monitoring [53]. These studies demonstrated that 11 respectively 15 blood pressure readings generated mean values that accounted for 80% of the variation in any one single measure. Likewise, blood pressure readings performed on different days display large variability as reflected by mean standard deviations as high as 12/8 mmHg [54]. The regression to mean phenomenon is known to further dilute blood pressure measurement results.

The total range of variation observed for 24-hour mean in healthy pregnant women is even larger; 28 and 26 mmHg for SBP and DBP, respectively. This range of blood pressure variability is approximately three times larger when computed on the basis of individual blood pressure measurements instead of the 24-hour mean [55].

It has been estimated that use of a single blood pressure measurement to assist in diagnostic decision making would overdiagnose hypertension in 20–30% of cases and miss a third of those who truly have the disease [56,57].

The *white coat effect*, defined as an office blood pressure exceeding mean daytime ambulatory pressure by at least 20 mmHg systolic and/or 10 mmHg diastolic has been found in as many as 73% of treated hypertensive subjects. It may occur more frequently in women than in men [58] and it is virtually impossible to be diagnosed on clinical examination alone [59].

*White-coat hypertension *(defined as high blood pressure only in the medical care environment) is reported in as many as 20 to 35% of patients in whom hypertension is diagnosed [60,61] and in nearly 30% of pregnant women [62]. ABPM in elderly of Syst-Eur trial showed that blood pressure was on average 22 mmHg higher on conventional than on daytime ambulatory measurement [63]. Likewise, the ABPM in the same trial revealed that older patients have a general propensity toward hypotensive states of different aetiologies reflecting considerable diurnal blood pressure variability, with periods of hypotension alternating with hypertension. This translates into the well-known elderly's greater susceptibility to adverse drug events and calls for accurate diagnosis and appropriately tailored treatment.

Newer insights in this area, further emphasizing the tremendous complexity of hypertension *per se*, comes from a study comparing the long-term, office blood pressure measurements with 24-hour ambulatory blood pressure measurement in treated hypertensive patients [64].

The study by Clement et al. [64] showed that when office and ambulatory blood pressure are compared as to their impact on the long-term prognosis, ambulatory blood pressure predicts prognosis significantly better, even after adjustment for any associated risk factors.

Among several valuable findings of this study, the most notable is that patients whose mean 24-hour systolic pressure was 135 mmHg or higher, when they were receiving treatment, were nearly twice as likely to have a cardiovascular event as patients with a mean 24-hour systolic pressure of less than 135 mmHg, regardless of their office blood pressure values.

The use of 24-hour ambulatory blood pressure measurement seems to be a *sine qua non *condition for accurate diagnosis in cases with hypertension recently termed *reversed *or *masked *hypertension [65,66], in which the office pressure used to be lower than the ambulatory pressure. It was estimated that only in US might be as many as 10 million subjects having this type of hypertension [43]. The new entity requires special attention to be given in two different clinical scenarios:

1). Patients with high office pressure but with low ambulatory pressure (no more likely than normotensive persons to have a cardiovascular event); intensive treatment of these patients, exclusively on the basis of office blood pressure measurement and/or their labelling as having "resistant hypertension" (in absence of associated risk factors or established organ damage), is likely to be deleterious and, it may even lead to adverse drug events;

2). Patients with low office blood pressure but high ambulatory blood pressure (who are in the opposite extreme of the hypertension spectrum); apparently, these patients are "well controlled", however, in need of intensified treatment as they seem to be running a worse long-term prognosis.

## Consequences of errors in blood pressure measurement

The multiple sources of errors that may be encountered in the office blood pressure measurement emphasize the great degree of non-accurate, misleading results generated by such assessments in daily clinical practice. Two examples bellow highlight the consequences of only minimal biased assessments (e.g., ± 5 mmHg).

An overestimating systematic error of 5 mmHg would misclassify some 27 million people as being hypertensive rather than having normal-high blood pressure, i.e., prehypertension [67]. The individual and societal consequences of initiating drug treatment in this segment of the population might be huge and, eventually translate into a real public health burden. Namely, patients with a low absolute risk may be exposed to the potential side effects of a treatment for little or no therapeutic benefit.

Further, a systematic error of underestimating true blood pressure by 5 mmHg would mean that 21 million people would go untreated as their real hypertension would be labelled as normal-high blood pressure [67]. In this scenario, patients with high absolute risk who are misclassified as having controlled hypertension will have a higher risk of cardiovascular event than necessary.

Both these scenarios may occur with rather high probability in clinical practice given the current, widespread model of healthcare based on a dichotomous paradigm of "yes/no" decision making as to establishing an initial diagnosis, as to the need to investigate or not investigate or, to treat or not to treat, currently arbitrarily fixed for hypertension at 140/90 mmHg.

Amazingly, the scientific community seems to accept the particular outlook reflected by this dichotomous reasoning despite widespread awareness that the risk associated with increasing blood pressure is graded and continuous. It begins as low as at 115/75 mmHg [1] and increases gradually without, however, a particular threshold being known on the BP curve that might discriminate between *risk/no risk *circumstances [68].

The lack of such a threshold combined with the information derived from a multitude of clinical trials, convincingly demonstrating the benefits of treatment across all levels of blood pressure in Western populations (not only in hypertension) [69-71], have generated "*the lower the blood pressure*, *the better*" – treatment philosophy, applicable to the general population and advocated as such by the majority of current guidelines.

The <140/90 mmHg cut-off point (respectively <130/80 mmHg for those with compelling indications) have been chosen as treatment *goal*/*target *for pharmacologically treated hypertensives. These targets are supposed to be pursued aggressively with even three or four drugs if needed, in order to achieve "optimal" or "normal" BP in young, middle-aged, or diabetic subjects (below 130/80 mmHg), and at least "high-normal" BP in elderly patients (i.e., <140/90 mmHg) [72,73].

Unfortunately, current evidence indicates that most patients fail to achieve systolic blood pressure below 140 mmHg [74-76]. According to a survey of hypertension in 10 countries worldwide, the proportion of patients achieving a blood pressure target below 140/90 mmHg ranged from a maximum of 27% in the USA to a minimum of less than 3% in Zaire [77]. In clinical trials focusing hypertension treatment the control rate is around 6% [78].

Mancia and Grassi [79] pointed out that even in the case of ideal scenario whereby "patient compliance and physician's expertise were ensured, attaining systolic blood pressure control would neither frequently nor easily be obtained".

Additionally, common sense suggests that there may be a level at which the benefits of treatment are outweighed by its side effects [80]. Likewise, it is obvious that the absolute benefits gained differ substantially between elderly and middle aged people and those with or without pre-existing cardiovascular disease [81,82]. Appropriate assessment of these variables is a matter of judgement of the treating physician, based not on occasional blood pressure measurements but rather, on complex clinical decision making employed on a case-to-case basis [83-88].

The aforementioned major problems with accurately measuring blood pressure in day-to-day clinical practice for the purpose of diagnosis and treatment of hypertension, highlight the magnitude of the uncertainty range around the current blood pressure cut-off point (140/90 mmHg), consisting of huge number of people being misdiagnosed of having or not having hypertension.

Failure in both directions are regrettable but, in the context of currently increasing aggressive approach to hypertension mandated by most guidelines, overdiagnosis exposes people unnecessarily to considerable risk for adverse drug reactions (ADR).

## Poor treatment compliance rates – often a reflection of overdiagnosis?

Patients' low compliance rate with the prescribed medication is a widely acknowledged problem in hypertension treatment. Up to 50% of the patients quit the treatment they were given within the first year [89-92]. Adverse events with antihypertensive treatment are to a large extent dose related. More than 75% of these ADRs occur as such, at initiation of antihypertensive treatment [93,94]. In the commonly asymptomatic patients with recent diagnosed hypertension, even adverse events of trivial degree of intensity (mild headache, dizziness, etc.) can be perceived as interfering with their normal life. This reasoning has shaped treatment strategies during many years toward the *lowest effective dose *(of whatever agent had been used) at initiation of therapy, with the aim to maximize the likelihood of successful early treatment and improve the long-term compliance rate.

The task of the prescribing physician when attempting to stick to the *lowest effective dose *principle is, however not easy, as the labeling information at hand is often not enough informative for confident decision making. This is the case even when the doctors carefully take into account the known individual variations in drug response due to differences in age, weight, sex, ethnic background, state of health, concomitant medication use, and genetic polymorphism in drug metabolism.

The all too high dosage of a particular drug at launch seems to be inherent in the current drug development paradigm. Namely, high doses are selected during the early clinical trials phases with the purpose of assessing the agent's efficacy and less so its safety profile. Maximizing efficacy by preferential selection of higher doses may, obviously, be deleterious from safety point of view, particularly when such doses selected in early trials are used to prove efficacy in later phase trials and eventually are brought to the market once the drug is granted marketing authorization. Several studies have highlighted deviations in the post-licensing dose administration levels [95,96]. One study reported 115 instances of changes to the defined daily dose (DDD) between 1982 and 2000. Of these, about 60% were reductions relative to the initially designated DDD. Of some reason, cardiovascular drugs had the most DDD changes. In a similar study of changes to labeling instructions after licensing by the FDA, 79% of drugs underwent a reduction in drug dosage. Another study reported a 69% decrease in the length of time after marketing for the dosage change to occur [97]. The poor dosage selection predisposes to adverse drug events and consequent poor patient compliance.

The problem above is further compounded by the initial drug dosage guidance available for physicians in US [98] – the JNC guidelines and the Physicians' Desk Reference (PDR). The latter seems to be the preferred source of reference for some 90% of American physicians [99] while being used extensively also by consumers. A comparison of the two sources (the JNC VI versus the PDR editions of 1999 and 2000) has revealed large dose disparities in 23 out of 34 drugs (68%) in five frequently prescribed antihypertensives: ACEs, ARBs, BB, CCBs and diuretics. The PDR initial doses were at least 100% higher than the JNC VI doses except for chlorthalidone and a brand of metoprolol succinate. Furthermore, the PDR recommended use of lower initial doses in elderly in only 8 (18%) of 45 different drugs. This is, certainly, a real problem as hypertension is mostly prevalent among people above 60 years of age, known to have physiologically decreased hepatic and renal function and in whom clustering of multiple risk factors and/or associated morbidities require daily intake of several drugs. This constellation of factors makes the elderly vulnerable to first-dose reactions such as hypotension, dizziness, syncope, headaches, etc. Commonly, these ADR are dose-related [100,101] and are the leading cause of older patients discontinuing antihypertensive therapy (102).

The well-intended use by many physicians of a "half-dose" of a particular drug at initiation of treatment is hampered by the impossibility of dividing capsules, coated tablets and drugs with irregular format.

The "start low, go slow" principle, promoted constantly during the past decades as a stepwise approach to treating patients with hypertension, allows patients time to adjust psychologically to the fact that they have hypertension. A meta-analysis of 354 trials involving 56,000 participants showed that blood pressure reductions produced by the major classes of drugs at standard dose are similar, and that half the standard dose reduces efficacy by only 20% while more than halving side-effects [103-105].

Given the current prehypertension category, most "new hypertensive" patients are counselled as to lifestyle changes that either may fail or simply are not enough to prevent *passing *the threshold to "real" hypertension (>140/90 mmHg). The distress about having hypertension and possibly requiring life-long drug therapy may lead to development of anxiety symptoms that may be mistaken for ADR, which may lead to skipping doses or quitting treatment.

Worse than that is the case of those patients who, through the nature of their hypertension (e.g., white coat hypertension with blood pressure in excess of 20/10 mmHg above the cut-off point), are candidates for combination treatment from the start. Indeed, for these patients, or those whose blood pressure levels increase to these levels, the current guidelines recommend that consideration should be given to initiating (first-line) therapy with two drugs, either as separate prescriptions or in fixed low-dose combination. The main logic of these recommendations is simplicity of use of such combinations and their potential to considerably improve the blood pressure response rate while minimizing the incidence of adverse effects.

This might be a best evidence approach in patients with *sustained hypertension *diagnosed by ABPM but, it might be a high-risk approach in cases with *white coat hypertension*, not recognized by conventional office blood pressure measurement.

## Physicians' non-compliance with treatment guidelines

Proper diagnosis of hypertension is of paramount importance for successful implementation in the clinical practice of current treatment guidelines. Consequently, failure to do so by the prescribing physician community suggests that there might be difficulties with accurately diagnosing hypertension, a fact, ultimately resulting in poor control of hypertension.

As control of hypertension, defined as BP < 140/90 mm Hg had been conferred status of quality indicator in the Health Employer Data Information Set by the National Committee for Quality Assurance in US [106], the potential reasons for any setback regarding this indicator are exposed to careful research. It should be emphasized, however, that the choice of this particular blood pressure threshold is neither evidence-based nor universally accepted.

Fourteen out of 27 national hypertension societies represented at the 17^th ^world conference of Hypertension League Council held in Montreal in 1997 adopted the 140/90 mmHg cut-off point for hypertension diagnosis while the remaining 13 societies stayed with 160/95 mmHg [107].

Not surprisingly, a questionnaire survey among primary care physicians in US indicated that a significant proportion of them were reluctant to seek treatment goal below 140/90 mmHg while, in the same time, being pretty tolerant with mildly elevated SBP in older patients. These findings led the authors of the article [108] to make the statement that "physician behavior makes a significant contribution to the poor rates of hypertension control".

The situation is very similar in UK where an editorial by Campbell entitled: "Patients decide how low they go, not targets" [109], generated a wave of rapid responses reflecting the general discontent with current guidelines on hypertension [110].

Another issue of discontent among practicing physicians is the way results of clinical trials are presented, a fact that seems to impact the prescribing propensity of physicians and the patients' willingness to accept treatment [111].

The apparently small range between the two thresholds under debate (140/90 – 160/95 mmHg), encompasses the great majority of individuals with hypertension in whom most of the hypertension-related morbidity and mortality is recorded [112,113]. Overlapping this range, as a matter of fact, is the widespread uncertainty of the practicing physicians as to how confidently to diagnose the cases within this range, whether to initiate drug treatment in them or not and, if yes, whether the benefit of treatment will outweigh its potential risks.

Given that most of older hypertensive patients in general practices have a clustering of risk factors [3], there is high probability that in many cases a careful treatment approach by the physician will need to take in consideration the combined use of aspirin, a lipid lowering drug, several antihypertensive agents and, in may cases, an antidiabetic agent as well. The high probability of this scenario is generated by the NCEP III guidelines [114] that lowered the eligibility requirements for using statin drugs through moving the LDL-C threshold from = 4.1 mmol/L to = 3.3 mmol/L. This change translated into some 36 million people being in need for treatment, as compared with 15 million, before (a 140% increase) [115]. In addition to this, the results of ASCOT-LLT [116] and the ALLHAT-LLT [117] trials have expanded the relevance of lipid lowering treatment to the majority of hypertensive patients.

Obviously, it is difficult to find an all-fit formula for all these uncertainties; however, an emerging trend is to incorporate into the treatment management decision making the values and preferences of patients, clinicians and the general public [118-121]. This is certainly a process that is going on at the level of physician-patient relationship, unfortunately not always as transparent and well documented as it should be. The consequence is that the patient's choice [86-88] of, eventually, not be willing to accept treatment is not recorded as such, with the physician getting the blame of leaving the patient untreated, alternatively, insufficiently treated.

Philipps et al. [122] emphasized this problem as follows:

"Despite advances......, health care providers often do not initiate or intensify therapy appropriately during visits of patients with these problems. We define such behavior *clinical inertia *– recognition of the problem but failure to act on it".

"........achieving standard-of-care goals in only limited numbers of treated patients must be attributed either to therapeutic ineffectiveness or to clinical inertia. The attainment of treatment targets in clinical trials shows the effectiveness of present therapies for hypertension, elevated LDL cholesterol levels, and diabetes – leaving clinical inertia as the presumptive basis for treatment failure in many patients with these disorders".

The content of the first quote seems to ignore most of the sensitive aspects that normally occur in the patient-physician relationship and in the mutual decision making process of the two parts involved. Likewise, the second quote assumes generalization of the mean global estimates of clinical trials to the particular individual as a universal task to be fulfilled by all practicing physicians.

Indeed, epidemiologic data indicate that the long-term average BP is the best predictor for risk [123], however, short-term measurements of office BP, especially when they are near cut-off points for normal, all too often result in false-positive diagnosis of hypertension [124].

But, "physician behavior" and failure of clinicians to monitor blood pressure aggressively and institute pharmacotherapy when indicated were invoked as causes for poor control of hypertension already by the NHANES III, in 1997 [125]. From that gentle form of being reminded of their failures, practicing physicians have got to acquaint themselves gradually with the thought that they really deserved being blamed for "clinical inertia".

Most recently they could find out that they might be about to loose entirely what was left of their credibility in terms of diagnosing and treating hypertension.

In an article by Graves and Sheps in the American Journal of Hypertension [126], the authors convey their opinion on that: "Physicians do not measure BP well, and even if they do, the usefulness of their BP measurements is significantly compromised by the white coat effect".

This might certainly be true, and I also agree that something should be done, however, extreme solutions like: "It is clear from evidence trails that current practice of BP measurement is inadequate. Thus, the question is not whether physicians should or should not measure BP in the hypertensive patients; rather, it is how are we to replace the physician measurement with a higher quality BP measurement", might be too long far fetched.

(Wondering, whether this speedy progress might, some day, end up with doctors being disqualified to use a stethoscope!)

The authors answer the hypothetical question and suggest that "trained observers" or validated automated devices should be used. They acknowledge, however, that observer-dependent errors seen in "trained observers" (commonly nurses) are similar in type and magnitude to those seen in physicians [127].

The most recent AHA Scientific Statement [128] makes the justifiable remark that: "...physician blood pressure measurements should not be used exclusively in the routine management of the hypertensive patient". It goes on to say that: "With careful training even unpaid volunteers in large population surveys can measure blood pressure accurately [129]. However, even with the newer automated devices, the accuracy of the readings can be optimal only if all observers are appropriately trained and retrained and conscientious about using appropriate technique".

Eligibility to become "Observer" is dependent on a number of physical and cognitive competencies required to perform, assess and report the blood pressure measurement. The physical requirement includes vision, hearing and eye/hand/ear coordination (see Additional file 5).

Furthermore, observers are supposed to undergo careful evaluation as to their ability to assess "different types of bias, general technique, and the interpretation of the measurements including an understanding of the normal variability of blood pressure by time of day, exercise, timing of antihypertensive medications, etc. The observers should know how and when to communicate blood pressure readings gathered at home or other settings to the health care professional responsible for the care of the patient and management of hypertension".

A comprehensive model of retraining of all observers is suggested, whereby: "a central master trainer trains the site master trainers, and they in turn train the observers at each site. This model could be replicated within hospitals, ambulatory care settings, and community agencies".

The AHA document gives insight in the area of home/self-monitoring of blood pressure as a methodology that has the potential to "improve both therapeutic compliance and blood pressure control", according to two studies from mid 80s [130,131].

Despite the benefits of home blood pressure measurements – being more reliable than those recorded in the clinic setting – the Working Group on Blood Pressure Monitoring of the ESH neither recommend extensive use of the model, nor empowers patients to take action on the basis of the results [132]. Even without the empowerment of measuring blood pressure themselves, patients take the freedom to adjust their drug treatment or to quit it, without necessarily taking into consideration the level of blood pressure or the advice from a doctor. An estimated 50% of drugs prescribed for long term use are not taken because of concerns for side effects and/or drug dependency, a fact that powerfully impacts the overall compliance figures [133].

Furthermore, the main advantage of the home blood pressure monitoring – accurate readings – is not a guarantee of accurate reporting to the physician. This fact is highlighted in two studies employing measurement devices with memory. Patients using them were supposed to carefully record their blood pressure and to report the results to their physicians. More than half the subjects omitted or fabricated readings [134,135]. Supposedly, only the other halves of the patients were sufficiently well trained regarding: "information about hypertension, procedure for self monitoring, advice on equipment and its proper use, and interpretation of protocol and data" [136].

ESC advocates the home blood pressure monitoring for detecting white coat hypertension among patients with persistently raised clinic blood pressure, however, it emphasizes that a diagnosis requires confirmation with ambulatory monitoring.

## Conclusion

A widespread awareness seems to emerge as to the doubtful clinical relevance of the most used investigation in clinical practice – the blood pressure measurement.

As mentioned in this paper, a great number of factors are contributing to the more than 100-year old method being carefully scrutinized, primarily because of the biological, random fluctuations of the blood pressure variable and the white coat effect.

These two latter aspects, as well as many others (the greater predictive value of the SBP, rather that that of the DBP, the increasing cardiovascular risk parallel with rising BP from values as low as 115/75 mmHg, the results of a multitude of randomized clinical trials), have forged a big quantum leap in our understanding of hypertension and seem to bring about the need for radical change, a real paradigm shift in the way we see and deal with blood pressure and with hypertension.

Sadly enough, the realization that the classically office-based BP can, on no account, be relied upon for diagnosis and treatment purposes, has generated a wave of mistrust with the practicing physicians, commonly involved in the management of patients with hypertension.

At closer scrutiny, however, most of the reproaches directed toward physicians are exaggerated and many of the "solutions" suggested extreme. Moreover, the peculiar "physician behavior" might be a deliberate acting to account for the uncertainties related to the much debated BP range of 140/90 – 160/95 mmHg and for the failure of the current health care systems to generalize implementation of ABPM in clinical practice, at least for all "first diagnosis" and treatment decision making.

The second part of this paper will attempt to elaborate on these latter issues.

## Competing interests

The author(s) declare that they have no competing interests.

## Supplementary Material

Additional File 1 Guidelines for blood pressure measurement. (Adapted from Perloff et al.). Click here for file

Additional File 2 Factors that can interfere with accuracy of BP measurement (after McAlistar and Strauss). Click here for file

Additional File 3 Effects of routine activities on BP (adapted from Campbell et al.). Click here for file

Additional File 4 Indications for ABPM (JNC VI). Click here for file

Additional File 5 Physical and cognitive competencies required in "observers" certified to measure blood pressure (AHA Scientific Statement, 2004). Click here for file
