# Supplementary material for: Beyond the Evidence of the New Hypertension Guidelines. Blood pressure measurement – is it good enough for accurate diagnosis of hypertension? Time might be in, for a paradigm shift (I)
Source: Curr Control Trials Cardiovasc Med. 2005 Apr 6;6(1):6. doi: 10.1186/1468-6708-6-6 (PMC1087862; doi:10.1186/1468-6708-6-6)
Supplement: Additional File 1 — Guidelines for blood pressure measurement. (Adapted from Perloff et al.). [file 1468-6708-6-6-S1.doc]

| - Seat the patient in a quiet, calm environment with a bared arm resting on a standard table or other support so the midpoint of the upper arm is at the level of the heart. - Estimate the circumference of the bare upper arm at the midpoint between the shoulder and the elbow, by inspection or tape measure, and select an appropriate cuff. The bladder inside the cuff should encircle 80% of the arm. - Place the cuff so that the midline of the bladder is over the arterial pulsation, then wrap and secure the cuff snugly around the subject's bare upper arm. - The lower edge of the cuff should be 2.5 cm above the antecubital fossa where the head of the stethoscope is to be placed. - Inflate the cuff rapidly to 70 mm Hg and then by 10 mm increments while palpating the radial pulse. Note the reading at which the pulse disappears and subsequently reappears during deflation. - Place the low frequency head (bell) of your stethoscope over the brachial artery pulsation. - Inflate the bladder rapidly and steadily to a pressure 20-30 mm above the level previously determined by palpation, then allow the bladder to deflate at 2 mm/sec while listening for the appearance of the Korotkoff sounds. - As the pressure in the bladder falls, note the manometer readings at the first appearance of repetitive sounds (phase I), at the muffling of these sounds (phase IV), and when they disappear (phase V). As long as the Korotkoff sounds are audible, the rate of deflation should be no more than 2 mm per pulse beat. - After the last Korotkoff sound is heard, the cuff should be deflated slowly for at least another 10 mm to ensure that no further sounds are audible, and then rapidly and completely deflated; the subject should then be allowed to rest for 30 seconds. - The systolic (phase I) and diastolic (phase V) pressures should be recorded immediately, to the nearest 2 mmHg. - The measurement should be repeated after at least 30 seconds have elapsed, and the two readings averaged. In clinical situations additional measurements may be made in the same or opposite arm, in the same or an alternative position. - Multiple visits are needed before the diagnosis of hypertension can be established; their exact number and frequency will depend on how much the blood pressure is raised and whether there are other cardiovascular risk factors. |
| --- |
