# Supplementary material for: Beyond the Evidence of the New Hypertension Guidelines. Blood pressure measurement – is it good enough for accurate diagnosis of hypertension? Time might be in, for a paradigm shift (I)
Source: Curr Control Trials Cardiovasc Med. 2005 Apr 6;6(1):6. doi: 10.1186/1468-6708-6-6 (PMC1087862; doi:10.1186/1468-6708-6-6)
Supplement: Additional File 2 — Factors that can interfere with accuracy of BP measurement (after McAlistar and Strauss). [file 1468-6708-6-6-S2.doc]

| **Factor** | **Measured *vs* actual blood pressure** | |
| --- | --- | --- |
|  | **Systolic blood pressure** | **Diastolic blood pressure** |
| **Patient** | | |
| Talking | 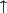 17 mmHg | 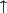 13 mmHg |
| Acute exposure to cold | 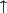 11 mmHg | 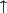 8 mmHg |
| Acute ingestion of alcohol | 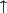 8 mmHg for 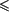3 hrs | 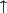 7 mmHg for 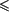3 hrs |
| **Technique** | | |
| Patient supine rather than  sitting | No effect;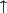 3 mmHg in supine position | 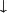 2-5 mmHg in supine position |
| Position of patient's arm | 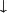 (or 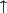) 8 mmHg for every 10 cm above (or below) heart level | 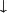 (or 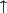) 8 mmHg for every 10 cm above (or below) heart level |
| Failure to support arm | 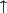 2 mmHg | 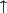 2 mmHg |
| Cuff too small | 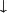 8 mmHg | 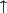 8 mmHg |
| **Measurer** | | |
| Expectation bias (including end digit preference) | Rounding to nearest 5 or 10 mmHg | Rounding to nearest 5 or 10 mmHg |
