# Supplementary material for: Beyond the Evidence of the New Hypertension Guidelines. Blood pressure measurement – is it good enough for accurate diagnosis of hypertension? Time might be in, for a paradigm shift (I)
Source: Curr Control Trials Cardiovasc Med. 2005 Apr 6;6(1):6. doi: 10.1186/1468-6708-6-6 (PMC1087862; doi:10.1186/1468-6708-6-6)
Supplement: Additional File 3 — Effects of routine activities on BP (adapted from Campbell et al.). [file 1468-6708-6-6-S3.doc]

| **Activity** | **Effect on blood pressure (mmHg)** | |
| --- | --- | --- |
|  | **Systolic blood pressure** | **Diastolic blood pressure** |
|  | | |
| Attending a  meeting | 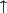 20 | 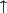 15 |
| Commuting to  work | 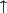 16 | 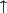 13 |
| Dressing | 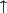 12 | 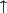 10 |
| Walking | 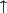 12 | 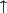 6 |
| Talking on  telephone | 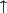 10 | 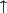 7 |
| Eating | 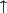 9 | 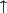 10 |
| Doing desk work | 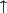 6 | 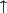 5 |
| Reading | 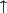 2 | 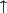 2 |
| Watching television | 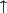 0.3 | 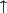 1 |
