# Supplementary material for: Beyond the Evidence of the New Hypertension Guidelines. Blood pressure measurement – is it good enough for accurate diagnosis of hypertension? Time might be in, for a paradigm shift (I)
Source: Curr Control Trials Cardiovasc Med. 2005 Apr 6;6(1):6. doi: 10.1186/1468-6708-6-6 (PMC1087862; doi:10.1186/1468-6708-6-6)
Supplement: Additional File 4 — Indications for ABPM (JNC VI). [file 1468-6708-6-6-S4.doc]

| - Suspected isolated hypertension - Autonomic dysfunction - Drug-induced orthostatic hypotension - Episodic hypertension - Resistant hypertension (BP > 140/90 or   > 160 mmHg for ISH despite triple-  drug regimen, including a diuretic) |
| --- |
