# Supplementary material for: Beyond the Evidence of the New Hypertension Guidelines. Blood pressure measurement – is it good enough for accurate diagnosis of hypertension? Time might be in, for a paradigm shift (I)
Source: Curr Control Trials Cardiovasc Med. 2005 Apr 6;6(1):6. doi: 10.1186/1468-6708-6-6 (PMC1087862; doi:10.1186/1468-6708-6-6)
Supplement: Additional File 5 — Physical and cognitive competencies required in "observers" certified to measure blood pressure (AHA Scientific Statement, 2004). [file 1468-6708-6-6-S5.doc]

| - **Vision**. The observer must be able to see the dial of the manometer ort he meniscus of the mercury column at eye level without straining or stretching, and must be able to read well enough to see the sphygmomanometer or digital display no further than 3 feet away. - **Hearing**. The observer must be able to hear the appearance and disappearance of Korotkoff sounds. - **Eye/hand/ear coordination**. This is required for the use of mercury and aneroid sphygmomanometers but not for the newer electronic technologies. |
| --- |
